# Apolipoprotein ε4 Is Associated with Lower Brain Volume in Cognitively Normal Chinese but Not White Older Adults

**DOI:** 10.1371/journal.pone.0118338

**Published:** 2015-03-04

**Authors:** Jennifer S. Yokoyama, Allen K. L. Lee, Leonel T. Takada, Edgar Busovaca, Luke W. Bonham, Steven Z. Chao, Marian Tse, Jing He, Christopher G. Schwarz, Owen T. Carmichael, Brandy R. Matthews, Anna Karydas, Michael W. Weiner, Giovanni Coppola, Charles S. DeCarli, Bruce L. Miller, Howard J. Rosen

**Affiliations:** 1 Department of Neurology, University of California San Francisco, San Francisco, California, United States of America; 2 Hospital das Clinicas, University of Sao Paulo Medical School, Sao Paulo, Brazil; 3 Department of Neurology, Veterans Affairs Health Care system, Palo Alto, California, United States of America; 4 Department of Neurology and Neurological Science, Stanford University School of Medicine, Stanford, California, United States of America; 5 Department of Neurology, University of California Davis, Davis, California, United States of America; 6 Department of Radiology, Mayo Clinic and Foundation, Rochester, Minnesota, United States of America; 7 Department of Computer Science, University of California Davis, Davis, California, United States of America; 8 Department of Neurology, Indiana University School of Medicine, Indianapolis, Indiana, United States of America; 9 Department of Veterans Affairs Medical Center, Center for Imaging of Neurodegenerative Diseases, San Francisco, California, United States of America; 10 Department of Radiology, University of California San Francisco, California, United States of America; 11 Departments of Psychiatry & Neurology, Semel Institute for Neuroscience and Human Behavior, University of California Los Angeles, Los Angeles, California, United States of America; University of Pennsylvania Perelman School of Medicine, UNITED STATES

## Abstract

Studying ethnically diverse groups is important for furthering our understanding of biological mechanisms of disease that may vary across human populations. The ε4 allele of apolipoprotein E (APOE ε4) is a well-established risk factor for Alzheimer’s disease (AD), and may confer anatomic and functional effects years before clinical signs of cognitive decline are observed. The allele frequency of APOE ε4 varies both across and within populations, and the size of the effect it confers for dementia risk may be affected by other factors. Our objective was to investigate the role APOE ε4 plays in moderating brain volume in cognitively normal Chinese older adults, compared to older white Americans. We hypothesized that carrying APOE ε4 would be associated with reduced brain volume and that the magnitude of this effect would be different between ethnic groups. We performed whole brain analysis of structural MRIs from Chinese living in America (n = 41) and Shanghai (n = 30) and compared them to white Americans (n = 71). We found a significant interaction effect of carrying APOE ε4 and being Chinese. The APOE ε4xChinese interaction was associated with lower volume in bilateral cuneus and left middle frontal gyrus (P_uncorrected_<0.001), with suggestive findings in right entorhinal cortex and left hippocampus (P_uncorrected_<0.01), all regions that are associated with neurodegeneration in AD. After correction for multiple testing, the left cuneus remained significantly associated with the interaction effect (P_FWE_ = 0.05). Our study suggests there is a differential effect of APOE ε4 on brain volume in Chinese versus white cognitively normal elderly adults. This represents a novel finding that, if verified in larger studies, has implications for how biological, environmental and/or lifestyle factors may modify APOE ε4 effects on the brain in diverse populations.

## Introduction

Ethnic diversity is important in medical research because differences in genetic background, environment and other sociocultural aspects (diet, language, access to care, etc.) may influence disease risk and manifestations. These differences have important implications for clinical management, particularly when there are established associations between ethnicity and risk for a disease as well as treatment response. Examples include genetic risk for isolated, late-onset cardiac amyloidosis in African Americans [1], gefitinib response in Japanese women with non-small cell lung carcinoma [2], and genetic contributions to asthma severity and bronchodilator response in admixed Hispanic populations [3–5]. An understanding of the differential effects of genetic factors across diverse populations and their effects on underlying biology is critical for furthering research and informing medical practice.

Apolipoprotein E ε4 (*APOE* ε4) is a well-known risk factor affecting the likelihood and age of onset of Alzheimer’s disease (AD) [6–12], with a dose dependence characteristic (*i*.*e*., two alleles are associated with an increased risk compared to one) [12,13]. Even in clinically normal elderly, there is evidence that *APOE* genotype affects likelihood of cognitive decline [14–16], and affects brain structure and function as measured by structural [17–19] and functional [20–25] neuroimaging, and neuropathology [26].

Frequency of the *APOE* ε4 allele varies across ancestral populations, with highest frequency in African populations (e.g., ~0.3 in Nigerians), medium frequency in European populations (e.g., ~0.14 in the UK) and lowest frequency in East Asian populations (e.g., ~0.07 in Chinese) [27]. The influence of *APOE* ε4 on AD varies across ethnic groups [6,28–31]. In addition to genetic differences across ethnicities, environmental and lifestyle factors also likely modulate how *APOE* ε4 alters the risk of AD. For example, Farrer, *et al*. [6] found that *APOE* ε4 showed weaker risk effects in African American and Hispanic individuals but stronger effects in Japanese when compared to white individuals.

Nearly 3.4 million ethnic Chinese live in America (in 2010 [32]), but they are still underrepresented in dementia studies [33,34]. In studies from China, *APOE* ε4 has been correlated with AD risk [7,35–37], as well as cognitive decline and memory performance in mild cognitive impairment (MCI) [38–40]. Neuroimaging studies found smaller hippocampal volumes in symptomatic ε4 carriers [35,41], but not among cognitively normal ε4-carrying controls [35]. Little to no research has been done to directly compare *APOE* ε4 effects between Chinese and white individuals.

In this study, we sought to investigate the role *APOE* ε4 genotype plays on brain structure in cognitively normal Chinese older adults, and to compare those patterns with a cohort of white Americans. We chose to study cognitively normal older adults for two reasons. First, structural changes in ε4 carriers—particularly in the hippocampal formation—may appear as early as infanthood [42] and adolescence [18], though measurable cognitive changes may only occur decades later [16]. Second, measures of cognitive impairment across diverse populations may be complicated by differences in language and culture [43,44]. By studying the baseline effects of *APOE* ε4 in older adults from ethnically diverse populations we could assess whether there are differential effects of ε4 on brain anatomy that may have implications for AD risk.

## Methods

### Subjects

All American participants were members of on-going studies in aging and cognition at the Memory and Aging Center (MAC) at the University of California, San Francisco (UCSF). Procedures for recruitment, enrollment, and for determining eligibility and ethnicity for Chinese Americans have been described in detail [33]. These participants are recruited through the Chinese Outreach portion of the MAC Alzheimer’s Disease Research Center (ADRC) from the San Francisco community through a variety of methods including: clinic assessments at the UCSF MAC and two clinic sites in Chinatown (the Chinatown Public Health Center and Chinese Hospital); lectures to local health care providers and community members; participation in community events; publications in mass media; word of mouth. The Chinese Outreach team consists of three bilingual and bicultural staff (one neurologist and two research assistants) that staff all research visits for Chinese Americans who speak primarily Chinese (Cantonese or Mandarin) or are bilingual Chinese/English. They translate all brochures and consent forms into Chinese and administer cognitive testing in Chinese. Chinese participants from Shanghai were recruited from the Jing’an district as described [41]. Study eligibility included diagnosis of clinically normal, available genetic information, and available MRI scans. White participants were selected to be as similar as possible to the Chinese (American + Shanghai) cohort with regard to age, sex, education, *APOE* ε4 allele distribution, and MR image acquisition field strength. For detailed inclusion and exclusion criteria see S1 Text. All subjects provided IRB-approved, written informed consent prior to participation, and all tests were approved by the University of California, San Francisco Committee on Human Research.

### Clinical Evaluation

All participants underwent a thorough multidisciplinary evaluation, including neurological exam, medical history, informant interview and cognitive testing. English-speaking research participants were tested using a standard battery to assess a broad range of cognitive domains including memory, executive and language [45,46]. The cognitive testing battery used at the MAC for Chinese speaking individuals is comprised of several measures validated for use in Chinese and others translated locally from English tasks to assess the same cognitive domains as the English-language battery. Research participation also included nursing evaluation that encompasses the Clinical Dementia Rating Scale (CDR [47]), and laboratory evaluation (described in [33,45]). Evaluation for Shanghai participants was administered as described^41^. For more details, see S1 Text.

### Image Acquisition and White Matter Hyperintensity (WMH) quantification

American participants underwent T1-weighted imaging using 1.5T (N = 4 Chinese, N = 4 white), 3T (N = 11 Chinese, N = 59 white), and 4T (N = 26 Chinese, N = 8 white) systems, with previously described sequences [48]. FLAIR image acquisition is provided in S1 Text. Automated WMH quantification in American participants was performed as described in Supporting Information [49]. Shanghai Chinese image acquisition and WMH quantification was performed as described [41]. Participants’ first available MRI was used for analysis and occurred within one year of their neuropsychological exam [41].

### Genotyping

Genomic DNA was extracted from peripheral blood using standard protocols (Gentra PureGene Blood Kit, QIAGEN, Inc.—USA, Valencia, CA). *APOE* genotyping (rs429358 and rs7412) was conducted using a TaqMan Allelic Discrimination Assay on an ABI 7900HT Fast Real-Time PCR system (Applied Biosystems, Foster City, CA) according to manufacturer’s instructions.

### Statistical Analysis

Two-tailed Pearson’s chi-squared analysis (nominal data) or ANOVA (interval data) was used to evaluate associations between demographic information or WMH volume and ethnic group byε4 carrier status. Two-tailed repeated measures ANOVA was used to test for significant change in neuropsychological test scores in Chinese participants for which longitudinal data was available. Analyses were performed in Stata/MP (v10.1, StataCorp LP, College Station, TX).

A standard image preprocessing and DARTEL warping pipeline was applied, and voxel-based morphometry (VBM) was performed on all structural MRIs using vlsm2.5 [50,51]. A general linear model (glm) was fit at each voxel (222,513 tests) to assess two primary effects: 1) the interaction of Chinese ethnicity with *APOE* ε4 carrier status (including main effects of ethnicity and ε4 carrier status) in all groups combined, and 2) ε4 carrier status as predictors of brain volume in the Chinese group separately. Secondary analyses tested the ε4 carrier effect in each of the three subgroups (US Chinese, Shanghai Chinese, Whites) to clarify the main effect in whites and rule out confounding due to country of residence in Chinese. Additional covariates in all models were age at time of scan, sex, total intracranial volume (TIV), scan type, and site of data collection. Detailed models are provided in S1 Text. VBM glms were one-tailed, assuming anti-correlation (*i*.*e*., carrying *APOE* ε4 is associated with smaller volume). Raw VBM results were examined at P_uncorrected_<0.001. Correction for multiple testing was performed using 1000 permutations on whole brain to determine the study-specific distribution of maximum T-scores and cluster sizes at a voxel-wise threshold of P_uncorrected_<0.001; family-wise error (FWE) corrected significance was established at P_FWE_≤0.05.

## Results

### Clinical characterization

A total of 142 images were analyzed from 41 Chinese Americans, 30 Shanghai Chinese, and 71 whites, with mean ages of 62, 72 and 68 years, respectively (P<0.0001, Table 1). There were a total of 31 *APOE* ε4-carriers as follows: eleven Chinese Americans (N = 10 with 1 allele, N = 1 with 2 alleles), 3 Shanghai Chinese (N = 3 with 1 allele, N = 0 with 2 alleles) and 17 whites (N = 17 with 1 allele, N = 0 with 2 alleles). Full genotype breakdown is provided in S1 Table. Mean education (in years) was significantly lower in Chinese individuals (P<0.0001, Table 1). Approximately 50% of the Chinese Americans were evaluated in Chinese (Mandarin or Cantonese).

**Table 1:** Sample demographics, by ethnic group and *APOE* ε4 carrier status.

		White (N_total_ = 71)		Chinese American (N_total_ = 41)		Shanghai Chinese (N_total_ = 30)		*P*
		ε4 (-)	ε4 (+)	ε4 (-)	ε4 (+)	ε4 (-)	ε4 (+)	
ε 4 Status	N	54	17	30	11	27	3	*ns*
Age	Mean ± SE	67.7 ± 1.2	68.5 ± 2.0	61.9 ± 1.9	62.8 ± 1.6	73.8 ± 1.2	72.3 ± 0.9	<0.001
	Range	39–83	53–83	44–82	47–75	65–88	71–74	N/A
Gender (Female)	%	67%	30%	70%	73%	48%	100%	<0.001
	Count	36/54	5/17	21/30	8/11	13/27	3/3	
Education	Mean ± SE	16.7 ± 0.3	17.2 ± 0.6	16.1 ± 0.6	15.2 ± 0.7	11.1 ± 0.9	4.5 ± 3.7	<0.001
	Range	11–20	12–20	9–20	8–20	0–20	0–9	N/A
English testing	%	100%	100%	40%	73%	0%	0%	<0.001
	Count	54/54	17/17	12/30	8/11	0/27	0/3	

Age and education are given as means ± standard error with range of values in parentheses. Statistics were performed across ethnic groups and by carrier ε4 status. N—sample size; age—age at time of image acquisition; ε4 (-)—no *APOE* ε4 alleles; ε4 (+)—*APOE* ε4 carriers (1 or 2 alleles); *P*—2-tailed p-value of association from Pearson’s chi-squared statistic for nominal traits (n, % female) or analysis of variance for interval (continuous) traits (age and education), where “*ns*” means not significant (*P*>0.05).

### Primary image analysis

**Fig 1:**
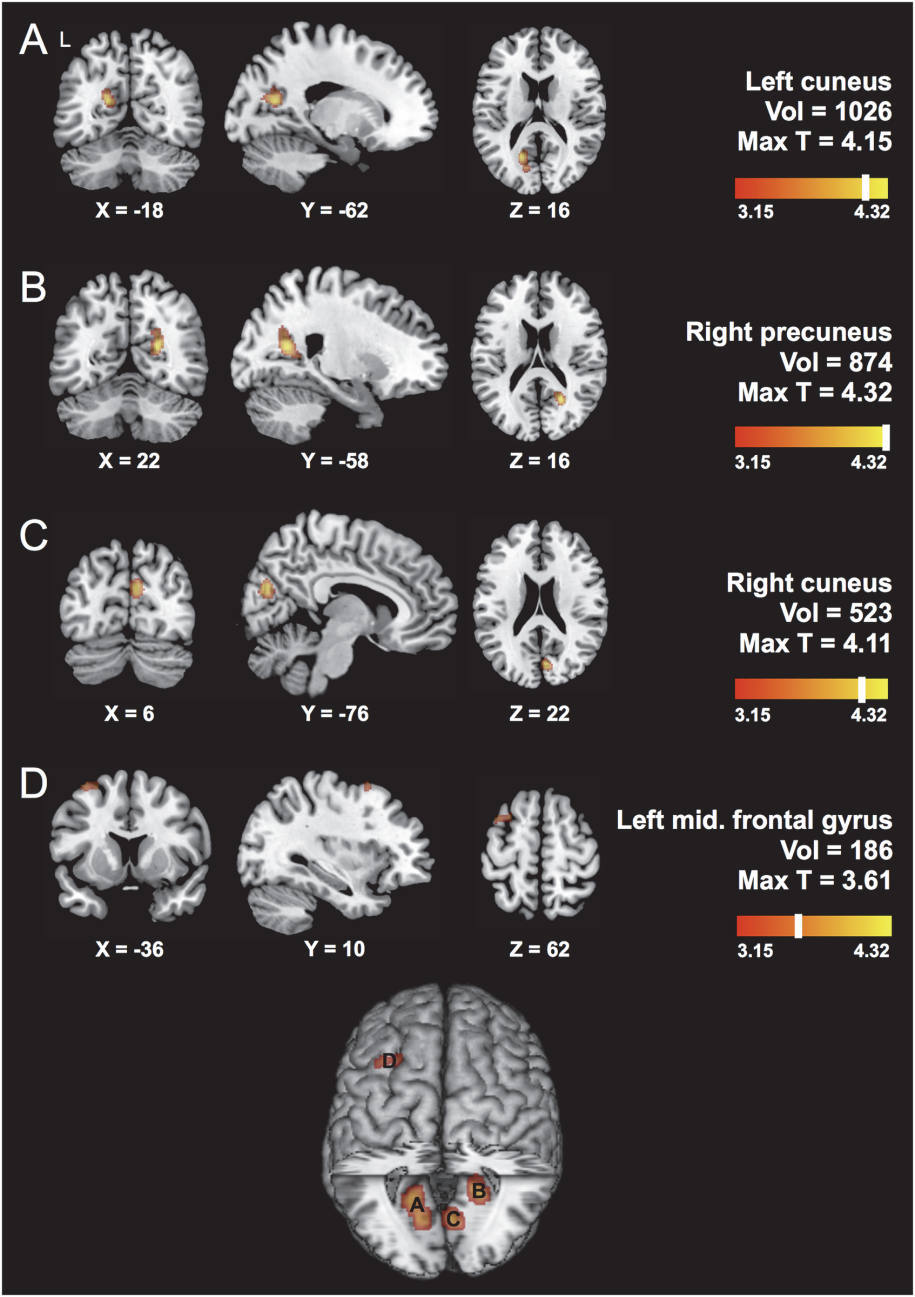
Interaction of *APOE* ε4 with Chinese ethnicity. Results from the interaction analysis of *APOE* ε4xChinese are shown for all individuals. Carrying *APOE* ε4 and being Chinese was associated with reduced volume in the (A) left cuneus, (B) right precuneus, (C) right cuneus and (D) left middle frontal gyrus (P_uncorrected_<0.001). Left cuneus remained significant after correction for multiple testing (P_FWE_ = 0.05). Regions are labeled according to the Automated Anatomical Labeling (AAL) Atlas, with volume (in mm^3^) and maximum T score provided for each cluster. Left side of image corresponds to left side of brain, with Montreal Neurological Institute (MNI) coordinates provided for respective slices. T-maps are shown at P_uncorrected_<0.001 (T range 3.15–4.32), overlaid on a template brain in MRICron. Single clusters were extracted using xjView toolbox (http://www.alivelearn.net/xjview). A summary of all findings is visualized on a rendered template brain in MRICron, with labels for each region as annotated above.

In the main analysis of our study, we tested whether there was a differential effect of *APOE* ε4 in Chinese versus white individuals. In the entire biracial cohort, the *APOE* ε4xChinese interaction was associated with lower volume in bilateral cuneus and left middle frontal gyrus (P_uncorrected_<0.001, Fig. 1, S2 Table). At a relaxed threshold of P_uncorrected_<0.01, there were also suggestive findings in right entorhinal cortex and left hippocampus (S1 Fig.). After correction for multiple testing, the left cuneus remained significantly associated with the interaction effect (P_FWE_ = 0.05). The interaction effect on these regions was, on average, two-fold the magnitude of the *APOE* ε4 main effect and 7-fold the magnitude of the main effect of race (S3 Table).

**Fig 2:**
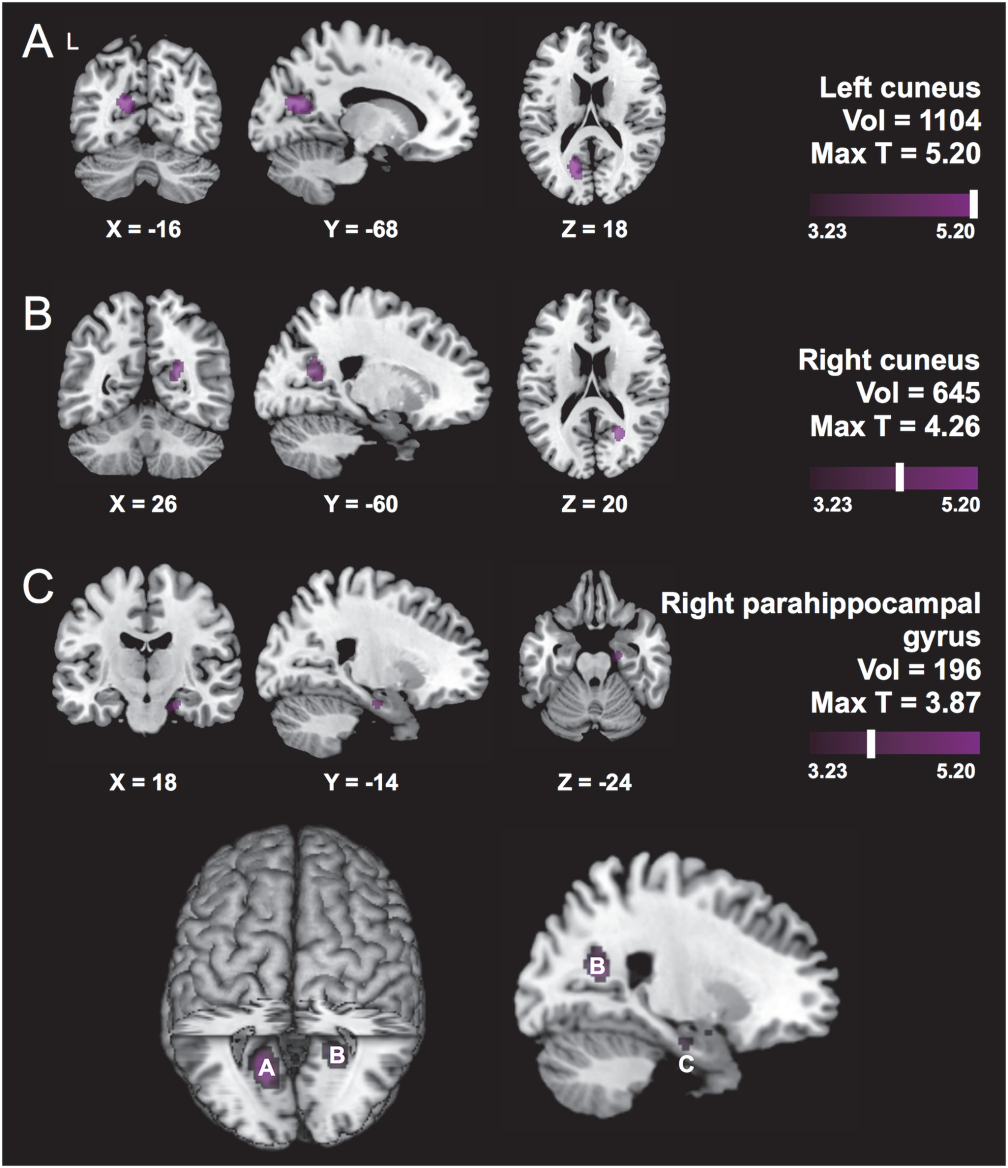
Main effect of carrying *APOE* ε4 on brain structure in Chinese. Results assessing the main effect of *APOE* ε4 in Chinese individuals only. Carrying *APOE* ε4 was associated with reduced volume in the (A) left cuneus, (B) right precuneus, and (D) right parahippocampal gyrus (P_uncorrected_<0.001). Left cuneus remained significant after correction for multiple testing (P_FWE_ = 0.04). Regions are labeled according to the Automated Anatomical Labeling (AAL) Atlas, with volume (in mm^3^) and maximum T score provided for each cluster. Left side of image corresponds to left side of brain, with Montreal Neurological Institute (MNI) coordinates provided for respective slices. T-maps are shown at P_uncorrected_<0.001 (T range 3.23–5.20), overlaid on a template brain in MRICron. Single clusters were extracted using xjView toolbox (http://www.alivelearn.net/xjview). A summary of all findings is visualized on a rendered template brain in MRICron, with labels for each region as annotated above.

**Fig 3:**
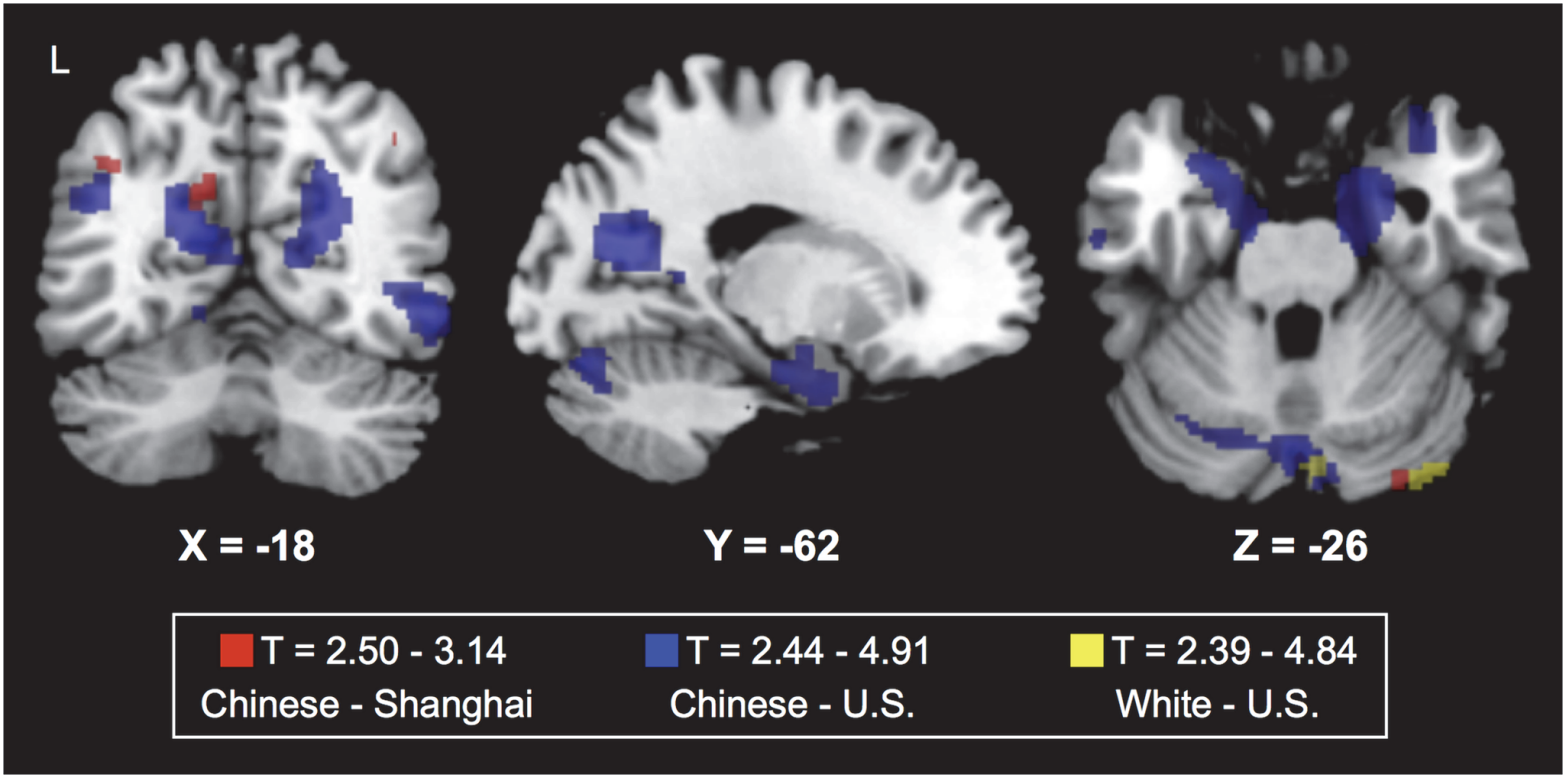
Main effect of carrying *APOE* ε4 on brain structure in Chinese and white subgroups. T-maps from cohort-specific analyses are shown at P_uncorrected_<0.01 overlaid on a template brain in MRICron. Whites are shown in yellow (T range 2.39–4.84), Chinese Americans in blue (T range 2.44–4.91), and Shanghai Chinese are in red (T range 2.50–3.14). Left side of image corresponds to left side of brain, with Montreal Neurological Institute (MNI) coordinates provided for respective slices. Both Chinese groups showed suggestive volume reductions in precuneus/cuneus in *APOE* ε4 carriers versus non-carriers. Chinese Americans also showed lower volume in the hippocampal formation.

To assess the main effects of *APOE* ε4 in Chinese, we next performed VBM in each ethnic group separately. In the Chinese cohort, *APOE* ε4 was associated with reduced volume in left cuneus, right precuneus, and right parahippocampal gyrus (P_uncorrected_<0.001, Fig. 2, S2–S3 Tables). After correction for multiple testing, reduced volume in the left cuneus remained significantly associated with carrying *APOE* ε4 (P_FWE_ = 0.04). In whites, *APOE* ε4 carriers only showed reduced volume in the cerebellum (P_uncorrected_<0.001; Fig. 3, in yellow; S4 Table).

## Secondary analysis of other possible confounds

To ensure that the *APOE* ε4 effect in the Shanghai participants was not markedly different than what we saw in the Chinese Americans, we performed secondary analysis on each Chinese subgroup separately. Chinese Americans and Shanghai Chinese but not whites both showed evidence of lower volume in cuneus and hippocampal formation (P_uncorrected_<0.01; Fig. 3, in blue and red; S4 Table) suggesting that the country a participant is living in is not a major confound.

We also assessed whether there were any baseline neuroanatomical differences between Chinese and white individuals by performing VBM on a subset of non- ε4 carriers that were matched for demographics. Our results showed differential neuroanatomic patterns consistent with speaking Chinese [52,53] or implicated in language and speech execution [54–56]. Importantly, none of the regions implicated in the APOE ε4 X Chinese interaction demonstrated changes in this *post hoc* VBM, suggesting that our primary findings are specific to being Chinese and carrying the ε4 risk allele. Refer to S1 Text for details.

Given previous association of *APOE* ε4 with WMH burden in cognitively impaired individuals [41], we assessed WMH volume in a subset of individuals for which FLAIR data was available to see if WMH volume was mediating the effect of *APOE* ε4 on brain volume in our cognitively normal Chinese groups. A total of 64 whites (N = 15 ε4 carriers, N = 49 non-carriers), 30 Chinese Americans (N = 9 carriers, N = 21 non-carriers) and 29 Shanghai Chinese (N = 3 carriers, N = 26 non-carriers) had WMH volumes available for analysis. WMH volume was significantly higher in Shanghai Chinese compared to whites (P<0.0001) and Chinese Americans (P<0.005), but there was no significant difference between the Chinese and white cohorts collected in the U.S. (S2 Fig.). Testing the main effect of ethnicity, WMH volume was not significant after adjusting for sample site (P = 0.58, t = 0.56). *APOE* ε4 was not a significant predictor of WMH volume across all ethnic groups (P = 0.41, F = 0.69) or after accounting for Chinese ethnicity (P = 0.46, t = -0.75). In the interaction model, WMH volume was significantly associated with age (P = 0.008, t = 2.70, beta = 0.17±0.06) and site (P = 0.007, t = -2.73) but not with the *APOE* ε4xChinese interaction (P = 0.68, t = -0.42), Chinese ethnicity (P = 0.56, t = 0.59), *APOE* ε4 carrier status (P = 0.85, t = 0.19), sex (P = 0.62, t = 0.50), TIV (P = 0.10, t = 1.68) or scan type (P = 0.60, t = 0.53). This suggested that WMH volume is not a mediator of the *APOE* ε4 effect specific to Chinese.

We also conducted all of the imaging analyses with education as a covariate because the Chinese American cohort had a significantly lower level of education (Table 1). The results did not differ from those without correction for education. Thus the possibility that education is mediating this effect is very unlikely.

Finally, in order to address the possibility that the Chinese participants were misclassified as cognitively normal, we assessed longitudinal data that was available for a subset of Chinese American samples collected at the MAC. Of 41 Chinese Americans, 32 returned for the first follow-up visit. Nine (22%) were only evaluated at one visit for the following reasons: six declined further participation and three were lost to follow-up. The attrition rate in the Chinese Americans was similar to that in the white participants, which was 23.9%. Cognitive assessment scores for the Chinese Americans returning for follow-up are shown in Table 2. Cognitive scores remained stable between Time 1 (baseline) and follow-up time points (repeated measures ANOVA, two-tailed P>0.05).

**Table 2:** Chinese American cognitive and functional scores over time.

	Time 1		Time 2		Time 3		*P*
**Chinese (Mandarin or Cantonese, N = 25)**	N		N		N		
CASI ± SD	25	96.1 ± 2.4	18	95.7 ± 2.6	13	97.0 ± 2.0	*ns*
MMSE ± SD	28	28.8 ± 1.1	18	28.4 ± 1.4	13	28.7 ± 1.1	*ns*
Verbal Fluency ± SD	20	15.5 ± 2.6	14	15.2 ± 2.0	13	17.7 ± 3.2	*ns*
Digits Forward ± SD	22	7.3 ± 1.0	17	7.5 ± 0.8	13	7.6 ± 0.7	*ns*
Digits Backward ± SD	22	5.7 ± 1.6	17	5.4 ± 1.5	13	5.6 ± 1.4	*ns*
**English, N = 8**							
MMSE ± SD	8	29.6 ± 0.5	6	29.0 ± 1.1	6	29.0 ± 1.7	*ns*
Verbal Fluency ± SD	8	20.4 ± 3.4	7	20.7 ± 5.7	6	22.8 ± 5.8	*ns*
Digits Forward ± SD	6	7.0 ± 0.6	5	6.2 ± 0.7	4	5.75 ± 0.8	*ns*
Digits Backward ± SD	8	5.0 ± 1.3	7	5.3 ± 1.3	6	5.5 ± 1.5	*ns*

Means with standard deviation and range are given for cognitive test scores at the time of the scan (Time 1), as well as subsequent clinic visits (Times 2–3). For Chinese language testing, cognitive measurements included the Chinese Cognitive Abilities Screening Instrument (CASI), CASI-derived Mini-Mental State Exam (MMSE), Verbal Fluency (Vegetable Naming), Digits Forward, and Digits Backward. For English language testing, cognitive measurements included the MMSE, Verbal Fluency (Animal Naming), Digits Forward, and Digits Backward. All cognitive scores were within normal limits. Two-tailed P-values are given for analysis of variance results, where “*ns*” means not significant (*P*>0.05).

## Discussion

In this study, we present results for a gene-ethnicity interaction effect on brain anatomy in cognitively normal older adults. We found an association of the *APOE* ε4 allele with volume loss in bilateral cuneus, with suggestive findings in parahippocampal gyrus, entorhinal cortex and hippocampal formation, which was only demonstrable in Chinese participants. The entorhinal cortex and hippocampus are among regions most associated with degeneration in AD, and have been suggested to be an early site of atrophy associated with *APOE* ε4 [18,42]. The cuneus has also been implicated in conversion and progression of AD [57,58]. Taken together, our results are consistent with atrophy patterns associated with AD, for which *APOE* ε4 is a known risk factor.

As discussed below, a gene-atrophy association for *APOE* ε4 has been demonstrated previously; however, to our knowledge, this is the first time that the ε4-carrier effect on neuroanatomy has been stronger in one ethnic group compared with another. This finding is consistent with several studies that have identified a differential AD risk effect of *APOE* ε4 across diverse populations [6,28–31]. However, whether genotype-associated differences in neuroanatomy directly reflect disease risk remains unclear. For example, previous work has shown alterations in connectivity in the functional network most affected by AD in *APOE* ε4 carriers versus non-carriers; these differences were observed in the absence of differences in gray matter volume in adolescent and young adults [59,60], and in older individuals in the absence of cognitive symptoms or amyloid deposition in the brain [61]. There could be baseline effects of *APOE* ε4 on the brain that alter its vulnerability to disease but are modified by other factors such as cognitive reserve. The fact that Chinese *APOE* ε4 carriers have volume loss in AD-associated brain regions yet maintain normal cognition is intriguing and, if verified in larger studies, suggests that biological, environmental, and/or lifestyle modifiers specific to Chinese may promote cognitive protection.

Two possible biological explanations for the present findings will be discussed. One possibility is that Chinese carriers are more susceptible to disease due to preclinical changes in brain structure associated with carrying *APOE* ε4. This would suggest that biological and/or environmental components moderate the *APOE* ε4 effect in this population, resulting in greater volume loss in AD-specific brain regions, which could result in higher risk to Chinese ε4 carriers of developing dementia later in life. However, longitudinal follow-up in our cohort of Chinese Americans suggested no evidence of decline in cognitive performance in 2–3 years of follow-up.

An alternative–and perhaps more intriguing–hypothesis is that Chinese *APOE* ε4 carriers are resistant to the risk for dementia conferred by the ε4 allele based on the presence of modifying factors specific to this population. This would be consistent with the fact that the penetrance of ε4 is not complete; there are some individuals who have two copies of the risk allele but never develop AD [11]. It is possible that the white ε4 carriers in our cohort also had volume loss in AD regions and subsequently advanced to MCI or AD status, such that the remaining white ε4 carriers in our cognitively normal cohort will continue to maintain their cognitive status for the duration of their life. In contrast, in Chinese there may be some form of moderation or compensation that allows maintenance of cognitive ability despite presence of ε4-associated volume loss, thereby delaying or preventing onset of dementia as compared to similarly at-risk whites. For example, Chinese individuals may demonstrate the normal effect of *APOE* ε4 in memory-specific systems, but other brain systems could be compensating for these changes; this could take the form of enhanced brain function in particular cognitive domains, or lifestyle factors that bolster cognitive reserve. Further study to identify modifiers that protect Chinese from cognitive decline despite *APOE* ε4-conferred loss in brain volume could provide a unique opportunity to identify factors that promote cognitive resilience against AD.

There are some caveats to the present study. In addition to a limited sample size, many of the Chinese American participants were evaluated with the Chinese language version of our neuropsychiatric assessment, which is not directly comparable to the English language evaluation administered to English-speaking Chinese Americans. Thus it is possible that Chinese-speaking individuals may have subtle impairments in cognitive performance that would have been detected by our English testing battery. However, the proportion of English tests administered to the Chinese American *APOE* ε4 carriers versus non-carriers (73% and 40%, respectively) is not significantly different, so any discrepancies related to testing language are likely balanced between the two genotype groups. Another potential confound is associated with the ages in the study groups. It is possible that the white cohort—with a mean age of 68 years of age—has simply had more time to convert to cognitive impairment as compared to the slightly younger (mean age 62) Chinese American subgroup. The Shanghai Chinese subgroup was older than both American groups (mean age 72); the low prevalence of *APOE* ε4 carriers in this group may be indicative that those who were going to convert to MCI/AD have already done so, though longitudinal data detailing this was not available for the present study. Age was included in the analysis as a covariate, so our findings should not be mediated by age, though better matching of groups would be ideal. In addition, there was no significant difference in age between *APOE* ε4 carriers versus non-carriers (Chinese American: P = 0.07; Shanghai Chinese: P = 0.19; white American: P = 0.10), further suggesting that the genotype effects we observed are not age-dependent. Finally, the present study was not powered to test whether the *APOE* ε4 effect we have observed applies equally to Chinese living in America and in China. Future studies including a larger number of ε4 carriers will be required to address this open question.

We assessed WMH volume as a potential confounder for the *APOE* ε4xChinese interaction but found no association of WMH volume with genotype or ethnicity after controlling for site of sample ascertainment. The Shanghai Chinese subgroup demonstrated significantly higher WMH volume than both American groups, even after controlling for age, consistent with previous findings [41]. Given the consistency of structural findings in both Chinese subgroups despite differences in WMH burden, it appears that WMH does not mediate this genotype-ethnicity effect. A limitation of this analysis was that WMH was quantified using two different methods, which may not be directly comparable. Future studies measuring all individuals with the same neuroimaging and processing protocol will allow for more thorough investigations of neuroanatomical mediators of *APOE* ε4 effects.

Another question arises regarding why we did not find any effect of *APOE* ε4 in our white cohort. Previous studies of the preclinical effect of ε4 on neuroanatomy have been inconsistent (reviewed in [20]); although many groups have found anatomical differences in cognitively normal youth and adults based on ε4 carrier status [18,42], others have not [17,25,62]. These negative findings include two studies of N = 164 [17] and N = 198 [62] predominantly white healthy controls from the well-characterized Alzheimer’s Disease Neuroimaging Initiative (ADNI) database (for more information see www.adni-info.org/). Factors that might influence the ability to detect this effect in normal elderly include the approach used to classify individuals as cognitively normal, as well as other co-morbid genetic and lifestyle factors that may influence the characteristics of the cohort.

In summary, we have demonstrated an *APOE* ε4 relationship to brain anatomy in cognitively normal older adults with a stronger effect in Chinese individuals. A critical next step will be to evaluate an independent cohort of Chinese and whites for replication of this finding. This will require expanding recruitment efforts to collect more samples and imaging data from diverse populations as well as expanding international collaborations, along with development of uniform approaches to clinical assessment. Longitudinal assessment will be key to evaluate the implications of any differences identified across ethnicities. This work has great potential for furthering our understanding of the factors contributing to risk for dementia across races and cultures.

## Supporting Information

S1 Table Genotypes Summarized by Sub-Group. Genotypes for individuals are summarized for each possible combination APOE ε allele type.
(DOCX)

S2 Table Results of Primary Analyses by Voxel-Based Morphometry. Results for the two primary analyses by voxel-based morphometry are displayed above. Regions are labeled according to their placement within the Automated Anatomical Labeling (AAL) atlas of the human brain. For each finding, the volume of the cluster and coordinates of the voxel within the cluster with the highest T-score are provided as X, Y, and Z values in the MNI152 coordinate system. Finally, the maximum T-score within each cluster, unadjusted P-value, and corrected P-value are provided. L—Left. R—Right.
(DOCX)

S3 Table Regression Results for Regions of Interest Found in Primary Analyses. After discovering seven regions of interest (ROI) by voxel-based morphometry, we confirmed our results by linear regression analyses. A regression was performed for each ROI. For the four ROIs found in the Chinese X *APOE* ε 4 interaction analysis, we used age, sex, total intracranial volume (TIV), scan type, race, *APOE* ε 4 carrier status, and *APOE* ε 4 X Chinese status as independent variables. For the three ROIs found in the all Chinese *APOE* ε 4 main effect analysis, we used age, sex, TIV, scan type, and *APOE* ε 4 carrier status as independent variables. The regression coefficient (Coef., ß), standard error (Std. Err.), and accompanying P-value are presented for each independent variable as predictors of ROI volumes. All tests were two-tailed. L—Left. R—Right.
(DOCX)

S4 Table Results of Secondary Analyses by Voxel-Based Morphometry. Results for the three secondary analyses by voxel-based morphometry are displayed above. Regions are labeled according to their placement within the Automated Anatomical Labeling (AAL) atlas of the human brain. For each finding, the volume of the cluster and coordinates of the voxel within the cluster with the highest T-score are provided as X, Y, and Z values in the MNI152 coordinate system. Finally, the maximum T-score within each cluster, unadjusted P-value, and corrected P-value are provided. L—Left. R—Right.
(DOCX)

S1 Fig Interaction of *APOE* ε4 with Chinese ethnicity. Results from the interaction analysis of *APOE* ε4xChinese are shown for all individuals. Left side of image corresponds to left side of brain, with Montreal Neurological Institute (MNI) coordinates provided for respective slices. T-maps are shown at the stated P-value thresholds, overlaid on a template brain in MRICron. Suggestive signal in hippocampal formation, with T-map shown at P_uncorrected_<0.01 (T range 2.35–4.32).
(DOCX)

S2 Fig White matter hyperintensity volumes. Mean±SE white matter hyperintensity volumes (in mm^3^) are shown for each subgroup, with whites in yellow, Chinese Americans in blue and Shanghai Chinese in red. **P<0.*005*, ***P<0.*0001* via 2-tailed Tukey-Kramer *post hoc* pair wise comparisons.
(DOCX)

S1 Text This file contains supporting methodological descriptions of cohort inclusion/exclusion criteria, clinical evaluation, FLAIR image acquisition, white matter hyperintensity quantification, and statistical analysis. It also contains a description of the secondary analysis of ethnicity as a confound.
(DOCX)
