# Supplementary material for: Apolipoprotein ε4 Is Associated with Lower Brain Volume in Cognitively Normal Chinese but Not White Older Adults
Source: PLoS One. 2015 Mar 4;10(3):e0118338. doi: 10.1371/journal.pone.0118338 (PMC4349764; doi:10.1371/journal.pone.0118338)
Supplement: S1 Table — Genotypes for individuals are summarized for each possible combination APOE ε allele type. (DOCX) [file pone.0118338.s001.docx]

**S1 Table: Genotypes Summarized by Sub-Group.**

|  | **All** | | **White** | | **US Chinese** | | **Shanghai Chinese** | |
| --- | --- | --- | --- | --- | --- | --- | --- | --- |
| **Genotype** | N | % | N | % | N | % | N | % |
| ε2ε2 | 1 | 1% | 1 | 1% | 0 | 0% | 0 | 0% |
| ε2ε3 | 16 | 11% | 7 | 10% | 3 | 7% | 6 | 20% |
| ε2ε4 | 2 | 1% | 1 | 1% | 0 | 0% | 1 | 3% |
| ε3ε3 | 94 | 66% | 46 | 65% | 27 | 66% | 21 | 70% |
| ε3ε4 | 28 | 20% | 16 | 23% | 10 | 24% | 2 | 7% |
| ε4ε4 | 1 | 1% | 0 | 0% | 1 | 2% | 0 | 0% |

**S1 Table Legend:** Genotypes for individuals are summarized for each possible combination APOE εallele type.
