# Supplementary material for: Apolipoprotein ε4 Is Associated with Lower Brain Volume in Cognitively Normal Chinese but Not White Older Adults
Source: PLoS One. 2015 Mar 4;10(3):e0118338. doi: 10.1371/journal.pone.0118338 (PMC4349764; doi:10.1371/journal.pone.0118338)
Supplement: S2 Table — Results for the two primary analyses by voxel-based morphometry are displayed above. Regions are labeled according to their placement within the Automated Anatomical Labeling (AAL) atlas of the human brain. For each finding, the volume of the cluster and coordinates of the voxel within the cluster with the highest T-score are provided as X, Y, and Z values in the MNI152 coordinate system. Finally, the maximum T-score within each cluster, unadjusted P-value, and corrected P-value are provided. L—Left. R—Right. (DOCX) [file pone.0118338.s002.docx]

**S2 Table: Results of Primary Analyses by Voxel-Based Morphometry.**

| **Chinese x *APOE* ε 4 interaction (thresholded at p<0.001)** | | | | | | |  |  |
| --- | --- | --- | --- | --- | --- | --- | --- | --- |
| **AAL Region** | **L/R** | **Volume (mm^3^)** | **X** | **Y** | **Z** | **Max T** | **Unadjusted *P*** | **Corrected *P*** |
| Cuneus | L | 1026 | -18 | -62 | 16 | 4.15 | <0.001 | 0.051 |
| Precuneus | R | 874 | 22 | -58 | 16 | 4.32 | <0.001 | 0.063 |
| Cuneus | R | 523 | 6 | -76 | 22 | 4.11 | <0.001 | 0.126 |
| Middle Frontal Gyrus | L | 186 | -36 | 10 | 62 | 3.61 | <0.001 | 0.318 |
| Inferior Frontal Gyrus – Pars triangularis | L | 14 | -42 | 22 | 0 | 3.24 | <0.001 | 0.651 |
| **All Chinese *APOE* ε 4 main effect (thresholded at p<0.001)** | | | | | | |  |  |
| **AAL Region** | **L/R** | **Volume (mm^3^)** | **X** | **Y** | **Z** | **Max T** | **Unadjusted *P*** | **Corrected *P*** |
| Cuneus | L | 1144 | -16 | -68 | 18 | 5.20 | <0.001 | 0.039 |
| Cuneus | R | 837 | 24 | -60 | 20 | 4.48 | <0.001 | 0.07 |
| Parahippocampal Gyrus | R | 219 | 20 | -14 | -22 | 3.61 | <0.001 | 0.31 |

**S2 Table Legend:** Results for the two primary analyses by voxel-based morphometry are displayed above. Regions are labeled according to their placement within the Automated Anatomical Labeling (AAL) atlas of the human brain. For each finding, the volume of the cluster and coordinates of the voxel within the cluster with the highest T-score are provided as X, Y, and Z values in the MNI152 coordinate system. Finally, the maximum T-score within each cluster, unadjusted P-value, and corrected P-value are provided. L – Left. R – Right.
