# Supplementary material for: Apolipoprotein ε4 Is Associated with Lower Brain Volume in Cognitively Normal Chinese but Not White Older Adults
Source: PLoS One. 2015 Mar 4;10(3):e0118338. doi: 10.1371/journal.pone.0118338 (PMC4349764; doi:10.1371/journal.pone.0118338)
Supplement: S3 Table — After discovering seven regions of interest (ROI) by voxel-based morphometry, we confirmed our results by linear regression analyses. A regression was performed for each ROI. For the four ROIs found in the Chinese X APOE ε 4 interaction analysis, we used age, sex, total intracranial volume (TIV), scan type, race, APOE ε 4 carrier status, and APOE ε 4 X Chinese status as independent variables. For the three ROIs found in the all Chinese APOE ε 4 main effect analysis, we used age, sex, TIV, scan type, and APOE ε 4 carrier status as independent variables. The regression coefficient (Coef., ß), standard error (Std. Err.), and accompanying P-value are presented for each independent variable as predictors of ROI volumes. All tests were two-tailed. L—Left. R—Right. (DOCX) [file pone.0118338.s003.docx]

**S3 Table: Regression Results for Regions of Interest Found in Primary Analyses.**

| **Chinese x *APOE* ε 4 interaction** | | | | | | | | | | | | | | | | | |
| --- | --- | --- | --- | --- | --- | --- | --- | --- | --- | --- | --- | --- | --- | --- | --- | --- | --- |
|  |  | **Age (years)** | | **Sex** | | **Total Intracranial Volume** | | **Scan Type** | | **Sample Site** | | **Race** | | ***APOE* ε 4 Carrier** | | ***APOE* ε 4 X Chinese Interaction** | |
| **AAL Region** | **L/R** | **Coef. ± Std. Err.** | ***P*** | **Coef. ± Std. Err.** | ***P*** | **Coef. ± Std. Err.** | ***P*** | **Coef. ± Std. Err.** | ***P*** | **Coef. ± Std. Err.** | ***P*** | **Coef. ± Std. Err.** | ***P*** | **Coef. ± Std. Err.** | ***P*** | **Coef. ± Std. Err.** | ***P*** |
| Cuneus | L | -5.88x10^-3^ ± 1.18x10^-3^ | 1.94x10^-6^ | -0.049 ± 0.022 | 0.03 | 4.01x10^-7^ ± 6.52x10^-8^ | 8.47x10^-9^ | 0.021 ± 0.022 | 0.34 | -0.19 ± 0.037 | 1.31x10^-6^ | 0.038 ± 0.028 | 0.18 | 0.070 ± 0.034 | 0.04 | -0.21 ± 0.050 | 4.67x10^-5^ |
| Precuneus | R | -4.51x10^-3^ ± 1.01x10^-3^ | 1.72x10^-5^ | -0.047 ± 0.019 | 0.01 | 4.36x10^-7^ ± 5.59x10^-8^ | 1.52x10^-12^ | 0.038 ± 0.019 | 0.05 | -0.029 ± 0.032 | 0.36 | 0.032 ± 0.024 | 0.19 | 0.067 ± 0.029 | 0.02 | -0.18 ± 0.043 | 3.69x10^-5^ |
| Cuneus | R | -5.54x10^-3^ ± 1.38x10^-3^ | 1.21x10^-4^ | -0.038 ± 0.026 | 0.16 | 3.80x10^-7^ ± 7.74x10^-8^ | 2.62x10^-6^ | 0.064 ± 0.026 | 0.02 | -0.070 ± 0.044 | 0.11 | 0.39 ± 0.034 | 0.25 | 0.17 ± 0.040 | 4.49x10^-5^ | -0.23 ± 0.060 | 1.58x10^-4^ |
| Middle Frontal Gyrus | L | -2.05x10^-3^ ± 7.42x10^-4^ | 6.59x10^-3^ | -6.48x10^-3^ ± 0.014 | 0.65 | 2.17x10^-7^ ± 4.11x10^-8^ | 5.12x10^-7^ | 0.026 ± 0.014 | 0.06 | 0.027 ± 0.023 | 0.25 | -6.02x10^-3^ ± 0.018 | 0.74 | 0.071 ± 0.021 | 1.06x10^-3^ | -0.12 ± 0.032 | 4.03x10^-4^ |
| **All Chinese *APOE* ε 4 main effect** | | | | | | | | | | | | | |  |  |  |  |
|  |  | **Age (years)** | | **Sex** | | **Total Intracranial Volume** | | **Scan Type** | | **Sample Site** | | **APOE ε 4 Carrier** | |  |  |  |  |
| **AAL Region** | **L/R** | **Coef. ± Std. Err.** | ***P*** | **Coef. ± Std. Err.** | ***P*** | **Coef. ± Std. Err.** | ***P*** | **Coef. ± Std. Err.** | ***P*** | **Coef. ± Std. Err.** | ***P*** | **Coef. ± Std. Err.** | ***P*** |  |  |  |  |
| Cuneus | L | -7.89x10^-3^ ± 1.48x10^-3^ | 1.36x10^-6^ | -0.014 ± 0.025 | 0.58 | 4.68x10^-7^ ± 8.22x10^-8^ | 3.55x10^-7^ | 0.016 ± 0.024 | 0.50 | -0.22 ± 0.035 | 1.42x10^-8^ | -0.14 ± 0.030 | 1.09x10^-5^ |  |  |  |  |
| Cuneus | R | -6.30x10^-3^ ± 1.37x10^-3^ | 2.02x10^-5^ | -0.011 ± 0.023 | 0.64 | 4.57x10^-7^ ± 7.59x10^-8^ | 9.43x10^-8^ | 0.024 ± 0.022 | 0.26 | -0.045 ± 0.032 | 0.16 | -0.13 ± 0.028 | 2.42x10^-5^ |  |  |  |  |
| Parahippocampal Gyrus | R | -5.42x10^-3^ ± 9.05x10^-4^ | 1.10x10^-7^ | -0.033 ± 0.015 | 0.03 | 3.33x10^-7^ ± 5.03x10^-8^ | 9.14x10^-9^ | 5.86x10^-3^ ± 0.015 | 0.69 | -0.041 ± 0.021 | 0.06 | -0.069 ± 0.018 | 3.39x10^-4^ |  |  |  |  |

**S3 Table Legend:** After discovering seven regions of interest (ROI) by voxel-based morphometry, we confirmed our results by linear regression analyses. A regression was performed for each ROI. For the four ROIs found in the Chinese X *APOE* ε 4 interaction analysis, we used age, sex, total intracranial volume (TIV), scan type, race, *APOE* ε 4 carrier status, and *APOE* ε 4 X Chinese status as independent variables. For the three ROIs found in the all Chinese *APOE* ε 4 main effect analysis, we used age, sex, TIV, scan type, and *APOE* ε 4 carrier status as independent variables. The regression coefficient (Coef., ß), standard error (Std. Err.), and accompanying P-value are presented for each independent variable as predictors of ROI volumes. All tests were two-tailed. L – Left. R – Right.
