# Supplementary material for: Apolipoprotein ε4 Is Associated with Lower Brain Volume in Cognitively Normal Chinese but Not White Older Adults
Source: PLoS One. 2015 Mar 4;10(3):e0118338. doi: 10.1371/journal.pone.0118338 (PMC4349764; doi:10.1371/journal.pone.0118338)
Supplement: S4 Table — Results for the three secondary analyses by voxel-based morphometry are displayed above. Regions are labeled according to their placement within the Automated Anatomical Labeling (AAL) atlas of the human brain. For each finding, the volume of the cluster and coordinates of the voxel within the cluster with the highest T-score are provided as X, Y, and Z values in the MNI152 coordinate system. Finally, the maximum T-score within each cluster, unadjusted P-value, and corrected P-value are provided. L—Left. R—Right. (DOCX) [file pone.0118338.s004.docx]

**S4 Table: Results of Secondary Analyses by Voxel-Based Morphometry.**

| ***APOE* ε4 Effect in US whites only (p<0.001)** | | | | |  |  |  |
| --- | --- | --- | --- | --- | --- | --- | --- |
| **AAL Region** | **L/R** | **Volume (mm^3^)** | **X** | **Y** | **Z** | **Max T** | **Unadjusted P** |
| Cerebellum | R | 267 | 2 | -82 | -26 | 4.84 | <0.001 |
| Cerebellum | R | 91 | 38 | -86 | -30 | 3.67 | <0.001 |
| Cerebellum | L | 20 | -36 | -88 | -28 | 3.37 | <0.001 |
| Superior Frontal Gyrus | R | 10 | 32 | 30 | 54 | 3.36 | <0.001 |
| ***APOE* ε4 effect in Chinese Shanghai only (p<0.005)** | | | | | |  |  |
| **AAL Region** | **L/R** | **Volume (mm^3^)** | **X** | **Y** | **Z** | **Max T** | **Unadjusted P** |
| Cuneus | L | 37 | -12 | -62 | 28 | 3.14 | <0.005 |
| Superior Frontal Gyrus | R | 24 | 20 | 46 | 20 | 2.99 | <0.005 |
| Inferior Occipital Gyrus | L | 17 | -30 | -84 | -10 | 2.98 | <0.005 |
| ***APOE* ε4 effect in US Chinese only (p<0.001)** | | | | |  |  |  |
| **AAL Region** | **L/R** | **Volume (mm^3^)** | **X** | **Y** | **Z** | **Max T** | **Unadjusted P** |
| Cuneus | L | 996 | -16 | -68 | 18 | 4.91 | <0.001 |
| Precuneus | R | 290 | 26 | -60 | 22 | 3.96 | <0.001 |
| Inferior Temporal Gyrus | R | 253 | 56 | -64 | -8 | 4.17 | <0.001 |
| Inferior Frontal Gyrus – Pars triangularis | L | 233 | -38 | 22 | -2 | 4.40 | <0.001 |
| Medial Superior Frontal Gyrus | L | 213 | 0 | 22 | 42 | 4.24 | <0.001 |
| Parahippocampal Gyrus | R | 115 | 16 | -14 | -24 | 3.82 | <0.001 |
| Insula | R | 81 | 38 | 26 | 2 | 3.55 | <0.001 |
| Temporal Pole | L | 74 | -28 | 8 | -36 | 3.56 | <0.001 |
| Middle Frontal Gyrus | L | 68 | 0 | -2 | 60 | 4.06 | <0.001 |
| Superior Frontal Gyrus | R | 27 | 14 | 60 | 36 | 3.61 | <0.001 |
| Cuneus | R | 27 | 24 | -42 | 8 | 3.77 | <0.001 |
| Cerebellum | R | 14 | 0 | -76 | -26 | 3.45 | <0.001 |
| Anterior Cingulate | L | 10 | -2 | 36 | 26 | 3.59 | <0.001 |

**S4 Table Legend:** Results for the three secondary analyses by voxel-based morphometry are displayed above. Regions are labeled according to their placement within the Automated Anatomical Labeling (AAL) atlas of the human brain. For each finding, the volume of the cluster and coordinates of the voxel within the cluster with the highest T-score are provided as X, Y, and Z values in the MNI152 coordinate system. Finally, the maximum T-score within each cluster, unadjusted P-value, and corrected P-value are provided. L – Left. R – Right.
