# Supplementary material for: Apolipoprotein ε4 Is Associated with Lower Brain Volume in Cognitively Normal Chinese but Not White Older Adults
Source: PLoS One. 2015 Mar 4;10(3):e0118338. doi: 10.1371/journal.pone.0118338 (PMC4349764; doi:10.1371/journal.pone.0118338)
Supplement: S1 Fig — Results from the interaction analysis of APOE ε4xChinese are shown for all individuals. Left side of image corresponds to left side of brain, with Montreal Neurological Institute (MNI) coordinates provided for respective slices. T-maps are shown at the stated P-value thresholds, overlaid on a template brain in MRICron. Suggestive signal in hippocampal formation, with T-map shown at Puncorrected<0.01 (T range 2.35–4.32). (DOCX) [file pone.0118338.s005.docx]

**
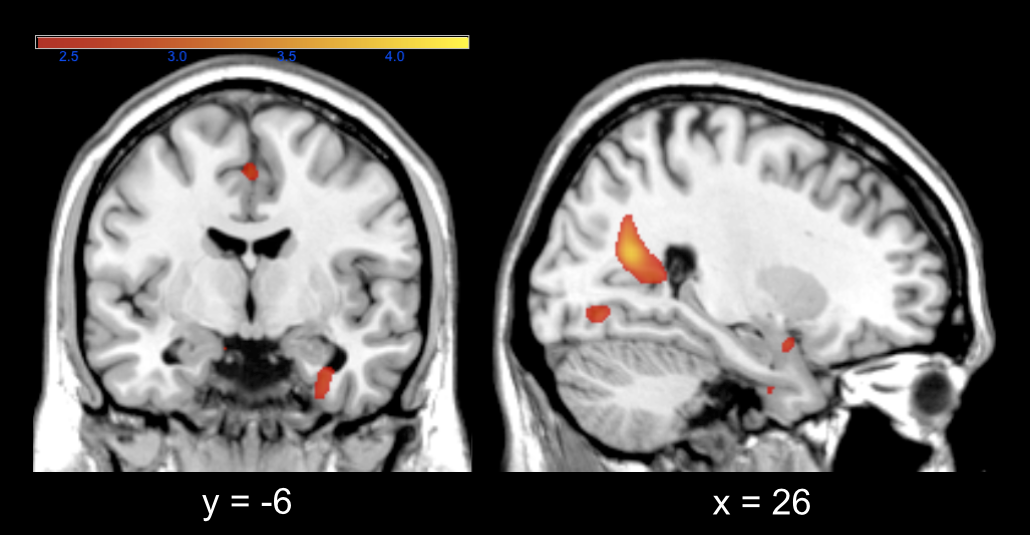
**

**S1 Figure: Interaction of *APOE* ε4 with Chinese ethnicity.** Results from the interaction analysis of *APOE* ε4xChinese are shown for all individuals. Left side of image corresponds to left side of brain, with Montreal Neurological Institute (MNI) coordinates provided for respective slices. T-maps are shown at the stated P-value thresholds, overlaid on a template brain in MRICron. Suggestive signal in hippocampal formation, with T-map shown at P_uncorrected_<0.01 (T range 2.35-4.32).
