# Supplementary material for: Apolipoprotein ε4 Is Associated with Lower Brain Volume in Cognitively Normal Chinese but Not White Older Adults
Source: PLoS One. 2015 Mar 4;10(3):e0118338. doi: 10.1371/journal.pone.0118338 (PMC4349764; doi:10.1371/journal.pone.0118338)
Supplement: S2 Fig — Mean±SE white matter hyperintensity volumes (in mm3) are shown for each subgroup, with whites in yellow, Chinese Americans in blue and Shanghai Chinese in red. **P<0.005, ***P<0.0001 via 2-tailed Tukey-Kramer post hoc pair wise comparisons. (DOCX) [file pone.0118338.s006.docx]

**S2 Figure: White matter hyperintensity volumes.** Mean±SE white matter hyperintensity volumes (in mm^3^) are shown for each subgroup, with whites in yellow, Chinese Americans in blue and Shanghai Chinese in red. ***P<0.005, ***P<0.0001* via 2-tailed Tukey-Kramer *post hoc* pair wise comparisons.
