# Supplementary material for: Apolipoprotein ε4 Is Associated with Lower Brain Volume in Cognitively Normal Chinese but Not White Older Adults
Source: PLoS One. 2015 Mar 4;10(3):e0118338. doi: 10.1371/journal.pone.0118338 (PMC4349764; doi:10.1371/journal.pone.0118338)
Supplement: S1 Text — It also contains a description of the secondary analysis of ethnicity as a confound. (DOCX) [file pone.0118338.s007.docx]

**Supporting Information**

**Methods**

*Inclusion and Exclusion Criteria.* Chinese Americans and white subjects included in this study had APOE genotypes available and were diagnosed as clinically cognitively normal by a multidisciplinary team of neurologists, neuropsychologists, and nurses within one year of image acquisition, based on review of the neurological history and examination, informant interview and cognitive testing. Exclusion criteria included psychiatric and/or neurological comorbidities; brain tumor; known positive HIV status; known pathogenic genetic mutations; use of benzodiazepines, opioids, steroids or other medications that could influence cognitive testing; and/or any factors that would prevent them from undergoing an MRI (e.g., metallic implant, claustrophobia). Participants were required to have an informant. Chinese from Shanghai were diagnosed as clinically cognitively normal as previously described by He, *et al.* [1].

*Clinical Evaluation.* All participants underwent a thorough multidisciplinary evaluation that begins with a neurological history and examination to identify if there are any cognitive complaints or findings on examination suggesting neurological disease and to evaluate the medical history. A separate interview with a knowledgeable informant is conducted to identify any concerns about the subjects’ cognition and the impact of these changes on function. Finally, all subjects had cognitive testing using a culturally and linguistically appropriate battery. All white participants were tested in English. Chinese American participants who were bilingual in English and Chinese (Cantonese or Mandarin) were asked what language they usually speak at home, and were tested in that language (N=20 English, N=21 Chinese). Neuropsychological assessment performed in Chinese included tests probing multiple domains of cognition, similar to the standard testing battery conducted in English: global cognitive function: the Chinese version of the Cognitive Abilities Screening Instrument (CASI [2]); verbal memory: one of two tests based on list-learning, the Chinese Version of the Verbal Learning Test (CVVLT [3]) or the Common Objects Memory Test (COMT [4]); executive function: Stroop Color Word Test [5], a design fluency task from the Ruff Figural Fluency Test (RFFT [6]) and Filled Dots trial of the Delis-Kaplan Executive Function System Design Fluency (D-KEFS DF [7]), Benson figure, digit span forward and backward, Color Trails Form A, trials 1 (CTT-1) and 2 (CTT-2) [8]; language assessment: category verbal fluency test (animals, vegetables). Mood was assessed using a translated version of the Geriatric Depression Scale Short Form [9,10]. Shanghai Chinese participants were tested as previously described [1].

*FLAIR Image Acquisition*. FLAIR images for American participants were acquired for individuals with 3T (N=56 white, N=6 Chinese American) and 4T (N=8 white, N=24 Chinese American) imaging only. 3T images were acquired at the Neuroscience Imaging Center at UCSF on a 3T Siemens MAGNETOM using T2-weighted Fluid Attenuated Inversion Recovery (T2 FLAIR) with the following parameters: TR/TE/TI 6000/388/2100 ms, 160 slices, slice thickness 1 mm, 250x250 matrix, and voxel size was 1x1x1 mm. 4T T2 FLAIR images were acquired at the Center for Imaging of Neurodegenerative Diseases at the San Francisco Veterans Affairs Medical Center on a Bruker MedSpec system controlled by a Siemens Trio console with the following parameters: TR/TE/TI 6000/405/2050 ms, 176 slices, slice thickness 1 mm, 256x224 matrix, and voxel size was 1x1x1 mm.

*White Matter Hyperintensity (WMH) Volume*. Fully-automated detection of WMH from coregistered T1- and FLAIR- weighted scans from American participants with all available images was performed using a previously-described method [11]. Briefly, all T1-weighted images were processed through the first six stages of the Freesurfer image analysis suite, which is documented and freely available for download online (http://surfer.nmr.mgh.harvard.edu/) to correct for intensity inhomogeneities [12] and create images stripped of non-brain tissue [13]; similarly, FLAIR images were processed through the “fast” utility of the FRMIB Software Library [14] (FSL, http://fsl.fmrib.ox.ac.uk/fsl/fslwiki/) to correct for scanner field bias gradient. Next, intra-subject FLAIR scans were rigidly coregistered to T1- and together nonlinearly transformed to a standard minimum-deformation-template space. In this space, the T1-weighted images were each aligned with intra-subject FLAIR images and the T1-weighted images with non-brain tissue (*i.e.*, voxels representing skull and connective tissue) removed were then used as a template to eliminate non-brain tissue in the FLAIR images. WMH voxels were detected by estimating a log-normal distribution of the multimodal image intensities for voxels in WMH- and non-WMH-probable image locations. These distributions comprised likelihood functions for Bayesian inference with an inhomogeneous Markov Random Field model incorporating both spatial and neighborhood-conditional priors. Resulting maps of detected WMH voxels were binarized by thresholding values above 0.2 by way of FSLmaths utilities then overlaid upon a white matter masks created individually for each subject based on the T1-weighted images; WMH voxels that were within the white matter mask of each subject were summed to estimate each subject’s global WMH volume. The methodology implemented to produce the initial WMH volume estimates have been previously validated to correlate strongly with those of a semi-automated approach in several large, diverse elderly cohorts [11].

*Statistical Analysis.* A general linear model (glm) was fit at each voxel (i) for the dependent variable of tissue volume (y_i_), and independent variables (x): ‘*APOE* ε4 carrier status by Chinese race’ (interaction), *APOE* ε4-carrier status (ε4-carrier), race (Chinese or not), age at time of scan (age), sex, total intracranial volume (TIV), scan type (scan) and whether data was collected at the MAC or in Shanghai (site).

For y_i_ = x*b we ran two models, where the variable of interest is underlined:

*(Model 1) y_i_ = interaction*b + ε4-carrier*b + race*b + age*b + sex*b + TIV*b + scan*b + site*b*

*(Model 2) y_i_ = ε4-carrier*b + age*b + sex*b + TIV*b + scan*b (+ site*b)*

Model 1 was run for the three cohorts combined including all Chinese participants as one group to examine the effect of ethnicity on the relationship between *APOE* ε4 and brain tissue (interaction). Model 2 was run in All Chinese (correcting for site) as well as separately for each subgroup (Chinese American, Shanghai Chinese, white).

**Results**

*Secondary analysis of other possible confounds.* To address if there were fundamental differences in brain anatomy between our cohort of Chinese Americans and whites that could confound the effect we observed of *APOE* ε4, we compared 28 Chinese American non-ε4 carriers to an age, sex, and education (in years) matched set of non-ε4 carrier whites using whole brain VBM (dummy variables: TIV, scantype). Our results showed neuroanatomic patterns consistent with previously identified regions related specifically to speaking Chinese [15]—including both Asians and European Chinese speakers [16]—or implicated in language and speech execution [17–19], where greater volume was observed in Chinese Americans as compared to whites in left inferior parietal region, left inferior temporal gyrus, left inferior frontal gyrus (pars triangularis), right thalamus, right supramarginal gyrus, and both right and left superior temporal poles. We also observed reduced volume in the cerebellum of Chinese Americans as compared to whites. Increased cerebellar volume has been shown for men versus women in both Chinese and Korean studies [20,21]; however, additionally controlling for sex in our Chinese American vs. white analysis did not remove this signal. Whether these findings represent biological differences related to ethnicity-specific brain morphometry or overall body size remains the topic of other studies. Importantly, none of the regions implicated in the *APOE* ε4 X Chinese interaction demonstrated changes in this *post hoc* VBM, suggesting that our primary findings are specific to being Chinese and carrying the ε4 risk allele.**References**

1. He J, Iosif A-M, Lee DY, Martinez O, Chu S, et al. (2010) Brain structure and cerebrovascular risk in cognitively impaired patients: Shanghai Community Brain Health Initiative-pilot phase. Arch Neurol 67: 1231–1237.

2. Teng EL, Hasegawa K, Homma A, Imai Y, Larson E, et al. (1994) The Cognitive Abilities Screening Instrument (CASI): a practical test for cross-cultural epidemiological studies of dementia. Int Psychogeriatr 6: 45–58; discussion 62.

3. Chang CC, Kramer JH, Lin KN, Chang WN, Wang Y-L, et al. (2010) Validating the Chinese version of the Verbal Learning Test for screening Alzheimer’s disease. J Int Neuropsychol Soc 16: 244–251.

4. Kempler D, Teng EL, Taussig M, Dick MB (2010) The common objects memory test (COMT): a simple test with cross-cultural applicability. J Int Neuropsychol Soc 16: 537–545.

5. Golden CJ (1978) Stroop Color and Word Test: A Manual for Clinical and Experimental Uses. Chicago, IL: Skoelting.

6. Ruff R (1996) Ruff Figural Fluency Test. Odessa, FL: Psychological Assessment Resources, Inc.

7. Delis DC, Kaplan E, Kramer JH (2001) The Delis-Kaplan Executive Function System. San Antonio, TX: The Psychological Corporation.

8. D’Elia L, Satz P, Uchiyama C, White T (1996) Color Trails Test. Odessa, FL: Psychological Assessment Resources, Inc.

9. Sheikh RL, Yesavage JA (1986) Geriatric Depression Scale (GDS). Recent Evidence and Development of a Shorter Version. Clin Gerontol 5: 165–173.

10. Lam CK, Lim PPJ, Low BL, Ng LL, Chiam PC, et al. (2004) Depression in dementia: A comparative and validation study of four brief scales in the elderly Chinese. Int J Geriatr Psychiatry 19: 422–428.

11. Schwarz C, Fletcher E, Decarli C, Carmichael O (2009) Fully-automated white matter hyperintensity detection with anatomical prior knowledge and without FLAIR. Lecture Notes in Computer Science (including subseries Lecture Notes in Artificial Intelligence and Lecture Notes in Bioinformatics). Vol. 5636 LNCS. pp. 239–251.

12. Reuter M, Rosas HD, Fischl B (2010) Highly accurate inverse consistent registration: A robust approach. Neuroimage 53: 1181–1196.

13. Smith SM, Jenkinson M, Woolrich MW, Beckmann CF, Behrens TE, et al. (2004) Advances in functional and structural MR image analysis and implementation as FSL. Neuroimage 23 Suppl 1: S208–S219.

14. Ségonne F, Dale AM, Busa E, Glessner M, Salat D, et al. (2004) A hybrid approach to the skull stripping problem in MRI. Neuroimage 22: 1060–1075.

15. Kochunov P, Fox P, Lancaster J, Tan LH, Amunts K, et al. (2003) Localized morphological brain differences between English-speaking Caucasians and Chinese-speaking Asians: new evidence of anatomical plasticity. Neuroreport 14: 961–964.

16. Crinion JT, Green DW, Chung R, Ali N, Grogan A, et al. (2009) Neuroanatomical markers of speaking Chinese. Hum Brain Mapp 30: 4108–4115.

17. Chesson AL (1983) Aphasia following a right thalamic hemorrhage. Brain Lang 19: 306–316.

18. Murdoch BE, Chenery HJ, Wilks V, Boyle RS (1987) Language disorders in dementia of the Alzheimer type. Brain Lang 31: 122–137.

19. Binder JR, Frost JA, Hammeke TA, Cox RW, Rao SM, et al. (1997) Human brain language areas identified by functional magnetic resonance imaging. J Neurosci 17: 353–362.

20. Chung SC, Lee BY, Tack GR, Lee SY, Eom JS, et al. (2005) Effects of age, gender, and weight on the cerebellar volume of Korean people. Brain Res 1042: 233–235.

21. Fan L, Tang Y, Sun B, Gong G, Chen ZJ, et al. (2010) Sexual dimorphism and asymmetry in human cerebellum: An MRI-based morphometric study. Brain Res 1353: 60–73.
